# The Occupational Depression Inventory performs well in Norway

**DOI:** 10.1038/s41598-026-52564-x

**Published:** 2026-05-08

**Authors:** Renzo Bianchi, Ingvild Andersen, Lars M. Rimol, Bendik Ola Larsen, Synøve Marie Gjaevran, Dorthe Bjergene Lyngbø, Kaja Bretteville Myhrvold, Allissa Mylan Nguyen, Thea Marie Otnes, Ada Fredrikke Hvidsten Roaldsøy, Gunnar Vade Sødal, Aurora Viggen, Maia Eriksen Zagury, Irvin Sam Schonfeld

**Affiliations:** 1 Department of Psychology, Norwegian University of Science and Technology (NTNU), Trondheim, Norway; 2 WorkWell Research Unit, North-West University, Potchefstroom, South Africa; 3 Oslo Business School, Oslo Metropolitan University (OsloMet), Oslo, Norway; 4 Department of Psychology, The City College of the City University of New York, New York City, NY USA

**Keywords:** Factor analysis, Mokken scaling, Occupational health, Work motivation, Workaholism, Workplace bullying, Workplace ostracism, Health care, Health occupations, Psychology, Psychology, Risk factors

## Abstract

This study examined the psychometric and structural properties of the Norwegian version of the Occupational Depression Inventory (ODI). Three independent samples of employees were recruited. Sample 1 included 382 individuals (50.8% female); Sample 2, 485 individuals (67.8% female); and Sample 3, 353 individuals (51.8% female). The ODI met the requirements for essential unidimensionality, strong scalability, and high total-score reliability. The instrument’s total score accurately ranked respondents on the latent dimension underlying the measure. The ODI showed a balance of convergent and discriminant validity when examined against a classical (i.e., an attribution-free) measure of depressive symptoms. Supporting the ODI’s criterion validity, occupational depression evinced a meaningful pattern of relations with work addiction, antidepressant intake, work motivation, physical assault at work, verbal abuse at work, workplace ostracism, sick leave, workplace bullying, and socioeconomic optimism. Work-attributed depressive symptoms were strongly linked to turnover intention. Measurement invariance held across samples, sexes, and age segments. The prevalence of occupational depression was estimated at 2.1% in Sample 1, 2.3% in Sample 2, and 2.0% in Sample 3. We conclude that occupational health specialists can rely on the ODI to assess job-related distress in the Norwegian working population.

**Supplementary Information:**

The online version contains supplementary material available at 10.1038/s41598-026-52564-x.

## Introduction

Norway has long stood out for its high level of sickness absence, with recent national statistics still placing total sickness absence at about 7% of agreed working time^1^. The rate of work absences in Norway exceeds that of other Nordic countries, which also have high absence rates relative to other OECD countries^1,2^. Norway’s public spending on sickness and disability benefits is about four times the OECD average^3^. Although Norway experiences substantial policy-level protection regarding work-related mental health and psychosocial hazards^4^, the share of employees who are sick-listed owing to mental health problems has been increasing for many years^5,6^. Mental health problems now account for over 25% of sickness-absence days^5^. With a 12-month prevalence estimated at 8%^7^, depression is the second most common mental disorder among Norwegians, only surpassed by anxiety^8^. Mental disorders top the list of the costliest conditions in Norway, with economic losses amounting to billions of US dollars/euros every year^9^. This context highlights the need for indicators of work-related mental health, notably of the distress associated with overwhelming job stress^10–12^.

The Occupational Depression Inventory (ODI) was introduced in 2020 as part of an effort to measure job-related distress more effectively^10,13^. The ODI was designed to assess depressive symptoms that individuals specifically ascribe to their job. In contrast to “classical” depression scales, which are purely symptom-centered, the ODI incorporates causal attributions to work (e.g., “My experience at work made me feel like a failure”). Causal attributions play an important role in how we interpret what happens to us and around us, subsequently influencing our actual emotions, motivations, cognitions, and actions^10,14,15^. For instance, attributing distress symptoms to toxic work relationships could prompt an employee to request sickness absence as a coping strategy to reduce immediate exposure to these stressors. Attributing a decline in mental health to a taxing and unsupportive work culture may push an individual to ultimately leave the organization to search for a healthier work environment or retire earlier than planned. Unsurprisingly, causal attributions lie at the heart of various work-contextualized constructs (e.g., work motivation and job-related affective well-being)^16,17^.

The ODI has been validated in multiple languages (e.g., English, French, Italian, Polish, [Brazilian-]Portuguese, Spanish, Swedish) and countries (e.g., the USA, Australia, New Zealand, South Africa, France, Switzerland, Italy, Poland, Brazil, Spain, Sweden) to date^18–26^. In terms of its factorial structure, the ODI has consistently exhibited essential unidimensionality. Essential unidimensionality refers to the property of a scale that, despite embodying a (small) degree of multidimensionality, can be considered a measure of a single phenomenon and used based on its total score^27^. The ODI has been assumed to be essentially unidimensional, rather than strictly unidimensional, on account of its anhedonic-somatic and dysphoric symptom items^10,13^. There is evidence of the ODI’s measurement invariance across countries, languages, sexes, and age segments^13^. In addition to displaying factorial validity, the ODI has demonstrated Mokken scale properties (e.g., strong scalability) and high total-score reliability^10,13,18–26^. Supporting the ODI’s criterion validity, occupational depression has been linked to various work and nonwork variables to which it was hypothesized to be linked. Such variables include, among others, demand-control discrepancy, effort-reward imbalance, general health status, objective cognitive performance, and company stock growth^10,21,22,25,28^. Finally, the ODI has shown a balance of convergent and discriminant validity when tested against (attribution-free) depression scales^10,20,24,25^. Such a balance is expected because, while unambiguously meant to assess depressive symptoms, the ODI specifically focuses on depressive symptoms that people attribute to their jobs^10,13^.

The present study examined the psychometric and structural properties of the Norwegian version of the ODI. More specifically, we inquired into the dimensionality, scalability, total-score reliability, criterion validity, convergent validity, discriminant validity, and measurement invariance of the measure. We opted for a multiple-sample approach to incorporate a self-replication component into the study design. Such a feature allows the generalizability of the findings to be more clearly established. We addressed criterion validity by inspecting the associations of occupational depression with an array of variables linked to depression in past research, including work addiction^29^, work motivation^30,31^, sick leave^32^, job promotion^33^, antidepressant intake^34^, physical assault and verbal abuse at work^19^, workplace ostracism^35^, socioeconomic optimism^36^, workplace bullying^37^, and turnover intention^10^.

The need for closer scrutiny of the measures developed in applied psychology has often been pointed out. As an illustration, Cortina et al.^38^ indicated that “[t]he distance between actual and recommended scale development and evaluation practices may have reached a magnitude that should lead us to question our conclusions regarding organizational phenomena…” (p. 1352). These authors further noted that “more rigorous validation may be required of a translated scale even if the process by which the original scale was developed was itself quite rigorous” (p. 1356). In a reexamination of widely employed psychological scales, Hussey and Hughes^39^ underlined the problem of hidden scale invalidity and the importance of conducting in-depth psychometric and structural scrutiny, preferably at an early stage of the measures’ development. The present study was conducted with these concerns in mind.

Based on the state of the art, we formulated five guiding hypotheses.

### Hypothesis 1

The Norwegian version of the ODI exhibits essential unidimensionality, strong scalability, and high total-score reliability.

### Hypothesis 2

Occupational depression correlates positively with work addiction, amotivation toward work, sick leave, antidepressant intake, physical assault at work, verbal abuse at work, workplace ostracism, workplace bullying, and turnover intention.

### Hypothesis 3

Occupational depression correlates negatively with work-related intrinsic motivation, job promotion, and socioeconomic optimism.

### Hypothesis 4

The Norwegian version of the ODI shows a balance of convergent and discriminant validity when tested against a classical, attribution-free measure of depressive symptoms.

### Hypothesis 5

The Norwegian version of the ODI demonstrates measurement invariance across samples, sexes, and age segments.

## Methods

### Study samples and recruitment procedure

Our study comprised three independent (i.e., non-overlapping) samples of participants recruited over the course of the year 2023. Each sample completed a specific survey (described below). All surveys included an attention-check item aimed at detecting careless responding (“For this question, please select the ‘somewhat agree’ option to show us that you are paying attention”). Participants’ age was assessed based on the following categories: 18–34 (early career); 35–49 (mid-career); and 50+ (late career)^40^. To be eligible for participation, individuals had to be (a) at least 18 years old and (b) currently employed.

Sample 1 was recruited with the help of the Nordic branch of the online survey company Bilendi (previously known as Respondi). Bilendi maintains proprietary online panels and recruits participants through multiple sources and selected industry partners. The company applies registration-level and ongoing quality-control procedures to ensure that panel members are real and unique, including checks designed to prevent bots, duplicate accounts, and false personal information. Bilendi invited panel members meeting the study’s prespecified eligibility criteria to complete our online survey. Bilendi’s services are widely relied on in academic research^41^. Of the 427 respondents who initially completed the survey, 45 (10.5%) were identified as inattentive and excluded, leaving a total of 382 participants. Of these 382 participants, 17.5% (*n* = 67) were aged 18–34, 40.1% (*n* = 153) were aged 35–49, and 42.4% (*n* = 162) were aged 50+; 50.8% of the respondents were female.

Sample 2 was recruited through contacts with local organizations (e.g., in the education and health sectors) and advertisements on social media (e.g., Facebook, LinkedIn). Of the 547 respondents who initially completed the survey, 62 (11.3%) were identified as inattentive and excluded, leaving a total of 485 participants. Of these 485 participants, 43.1% (*n* = 209) were aged 18–34, 24.7% (*n* = 120) were aged 35–49, and 32.2% (*n* = 156) were aged 50+; 67.8% of the participants were female.

Sample 3 was, like Sample 1, recruited with the assistance of Bilendi. Of the 400 respondents who initially completed the survey, 47 (11.8%) were identified as inattentive and excluded, leaving a total of 353 participants. Of these 353 participants, 26.9% (*n* = 95) were aged 18–34, 37.7% (*n* = 133) were aged 35–49, and 35.4% (*n* = 125) were aged 50+; 51.8% of the participants were female.

The study was conducted in accordance with the ethical principles of the Declaration of Helsinki, the Belmont Report, and the Norwegian Agency for Shared Services in Education and Research (Ref. 696536; https://sikt.no/en/home). The study involved anonymous data collection. Participation in the survey was voluntary. Respondents provided informed consent to participate. Bilendi, which helped us recruit two of the three samples, adheres to the General Data Protection Regulation, an information privacy regulation applied in the European Union and the European Economic Area.

### Measures

#### Occupational depression

Our main measure of interest was the ODI^10,13^. The ODI was developed with reference to the diagnostic criteria for major depressive disorder found in the *Diagnostic and Statistical Manual of Mental Disorders*, fifth edition (DSM-5)^14^. The instrument comprises nine symptom items rated on a frequency scale from 0 (“never or almost never”) to 3 (“nearly every day”). The scale covers the past two weeks. Consistent with the DSM-5, the symptoms assessed are anhedonia, depressed mood, sleep alterations, fatigue/loss of energy, appetite alterations, feelings of worthlessness, cognitive impairment, psychomotor alterations, and suicidal ideation. The ODI features a subsidiary question about turnover intention with the response options “no,” “I don’t know,” and “yes.” For analytical purposes, these response options can be recoded 1, 2, and 3, respectively. The ODI includes instructions for respondents on how to complete the scale. Respondents are asked to consider various sources of their symptoms–both related and unrelated to work–as well as the possibility of unknown sources. Respondents are invited to mark a symptom with a “0” if they believe the symptom is not caused by work or if they cannot identify the cause. The goal of these instructions is to prevent hasty imputations of symptoms to work.

The ODI addresses occupational depression through both a dimensional perspective, which views symptoms on a continuum from virtually absent to extremely severe, and a categorical perspective, using an algorithm that provides provisional diagnoses^10,13^. The algorithm is consistent with the DSM-5’s diagnostic criteria for major depression^14^. A provisional diagnosis is assigned when an individual scores 3 on at least five of the nine symptom items of the ODI, provided that one of these items is either anhedonia (item 1) or depressed mood (item 2). A score of 3 reflects experiencing symptoms “nearly every day,” aligning with the DSM-5’s indication that “the criterion symptoms for major depressive disorder must be present nearly every day to be considered present” (p. 162)^14^. Suicidal ideation (item 9) is treated differently. It contributes to the provisional diagnosis even when scored as 1 or 2–symptoms experienced “a few days only” or “more than half the days.” This exception accounts for the inherent seriousness of suicidality. The dual approach embodied in the ODI aligns with contemporary developments in psychopathological science that integrate dimensional and categorical perspectives. By using the ODI, investigators can estimate the severity of work-related depressive symptoms *and* identify probable cases of occupational depression.

We translated the ODI into Norwegian using a back-translation method^42^. First, the English version of the instrument was translated into Norwegian by two native Norwegian speakers fluent in English. Second, the Norwegian version of the instrument was translated back into English by a different native Norwegian speaker fluent in English. Third, the original and back-translated English versions were compared with one another. No problematic discrepancies were identified between the two versions. The items of the ODI translated into Norwegian are displayed in Table 1. The full Norwegian version of the instrument, including the instructions to respondents, is available in Supplemental Material 1. In addition, Supplemental Material 1 contains SPSS syntax that encapsulates the ODI’s provisional diagnosis algorithm. Descriptive statistics for the items of the ODI are provided in Table 2. The prevalence of occupational depression was estimated at 2.1% in Sample 1, 2.3% in Sample 2, and 2.0% in Sample 3.

**Table 1:** Norwegian version of the Occupational Depression Inventory (ODI).

Symptoms	Items
Anhedonia	Mitt arbeid var så stressende at jeg ikke kunne glede meg over ting jeg vanligvis liker å gjøre. *(My work was so stressful that I could not enjoy the things that I usually like doing.)*
Depressed mood	Jeg følte meg deprimert på grunn av jobben min. *(I felt depressed because of my job).*
Sleep alterations	Stress relatert til jobben førte til søvnproblemer (jeg hadde vanskelig for å sovne eller sove uforstyrret, eller jeg sov mye mer enn vanlig). *(The stress of my job caused me to have sleep problems [I had difficulties falling asleep or staying asleep*,* or I slept much more than usual].)*
Fatigue/loss of energy	Jeg følte meg utmattet på grunn av arbeidet mitt. *(I felt exhausted because of my work.)*
Appetite alterations	Jeg følte at appetitten min ble forstyrret på grunn av jobbstress (jeg mistet appetitten min, eller det motsatte, jeg spiste for mye). *(I felt my appetite was disturbed because of the stress of my job [I lost my appetite*,* or the opposite*,* I ate too much].)*
Feelings of worthlessness	Min opplevelse på jobb fikk meg til å føle meg mislykket. *(My experience at work made me feel like a failure.)*
Cognitive impairment	Jobben min stresset meg så mye at jeg hadde problemer med å fokusere på det jeg gjorde (f.eks. å lese en avisartikkel) eller å tenke klart (f.eks. å ta beslutninger). *(My job stressed me so much that I had trouble focusing on what I was doing [e.g.*,* reading a newspaper article] or thinking clearly [e.g.*,* to make decisions].)*
Psychomotor alterations	Som et resultat av jobbstress følte jeg meg rastløs, eller det motsatte, alt gikk saktere–for eksempel i måten jeg beveget meg eller snakket på. *(As a result of job stress*,* I felt restless*,* or the opposite*,* noticeably slowed down–for example*,* in the way I moved or spoke.)*
Suicidal ideation	Jeg tenkte at jeg ville heller være død enn å fortsette i denne jobben. *(I thought that I’d rather be dead than continue in this job.)*
Turnover intention (SQ)	Dersom du har støtt på minst noen av problemene nevnt ovenfor, fører disse problemene til at du vurderer å slutte i din nåværende jobb eller stilling? *(If you have encountered at least some of the problems mentioned above*,* do these problems lead you to consider leaving your current job or position? )*

The full ODI form (including the instructions to respondents) is available in Norwegian in Supplemental Material 1, together with an SPSS syntax implementing the provisional diagnosis algorithm of the ODI. SQ: subsidiary question.

**Table 2:** Descriptive statistics for the items of the Occupational Depression Inventory.

Indicators	ODI1	ODI2	ODI3	ODI4	ODI5	ODI6	ODI7	ODI8	ODI9
Sample 1 (*N* = 382)									
Mean	0.623	0.518	0.644	0.880	0.445	0.526	0.497	0.432	0.168
Median	0	0	0	1	0	0	0	0	0
Mode	0	0	0	1	0	0	0	0	0
Standard deviation	0.742	0.762	0.813	0.879	0.754	0.752	0.742	0.742	0.479
Skewness (*SE* = 0.125)	1.122	1.421	1.180	0.843	1.649	1.365	1.581	1.786	3.040
Kurtosis (*SE* = 0.249)	1.018	1.407	0.794	0.060	1.936	1.287	2.262	2.672	9.086
Minimum	0	0	0	0	0	0	0	0	0
Maximum	3	3	3	3	3	3	3	3	3
Sample 2 (*N* = 485)									
Mean	0.753	0.596	0.843	1.029	0.573	0.645	0.555	0.590	0.097
Median	1	0	1	1	0	0	0	0	0
Mode	0	0	0	1	0	0	0	0	0
Standard deviation	0.816	0.799	0.900	0.911	0.836	0.794	0.733	0.817	0.403
Skewness (*SE* = 0.111)	0.941	1.321	0.809	0.651	1.405	1.220	1.139	1.339	4.952
Kurtosis (*SE* = 0.221)	0.358	1.220	−0.239	−0.319	1.175	1.119	0.586	1.121	27.096
Minimum	0	0	0	0	0	0	0	0	0
Maximum	3	3	3	3	3	3	3	3	3
Sample 3 (*N* = 353)									
Mean	0.691	0.530	0.805	1.042	0.484	0.544	0.490	0.445	0.176
Median	1.000	0.000	1.000	1.000	0.000	0.000	0.000	0.000	0.000
Mode	0	0	0	1	0	0	0	0	0
Standard deviation	0.779	0.776	0.855	0.876	0.816	0.790	0.765	0.725	0.546
Skewness (*SE* = 0.130)	0.999	1.368	0.852	0.554	1.678	1.420	1.544	1.609	3.549
Kurtosis (*SE* = 0.259)	0.574	1.122	0.005	−0.352	1.980	1.390	1.745	1.978	12.977
Minimum	0	0	0	0	0	0	0	0	0
Maximum	3	3	3	3	3	3	3	3	3

SE = standard error; ODI1: anhedonia; ODI2: depressed mood; ODI3: sleep alterations; ODI4: fatigue/loss of energy; ODI5: appetite alterations; ODI6: feelings of worthlessness; ODI7: cognitive impairment; ODI8: psychomotor alterations; ODI9: suicidal ideation. There were no missing values.

#### Sick leave and job promotion

Participants indicated whether they had been (a) on sick leave and (b) promoted in their job (as reflected in higher status/income) over the past six months. Response options were “yes” and “no.” Sick leave and job promotion were assessed in all samples.

#### Addiction to work

We used the Bergen Work Addiction Scale (BWAS) to assess addiction to work^43^. The BWAS comprises seven items (e.g., “Thought of how you could free up more time to work?”) rated from 1 for “never” to 5 for “always.” The developers of the BWAS suggest that a score of at least 4 (“often” or “always”) on at least 4 of the 7 BWAS items differentiates workaholics from non-workaholics. McDonald’s omega was 0.856 and Cronbach’s alpha was 0.855. Addiction to work was assessed in Sample 1.

#### Work motivation

Work motivation was assessed with the Multidimensional Work Motivation Scale (MWMS)^16^. We specifically focused on two motivational aspects, namely, intrinsic motivation (McDonald’s omega = 0.905; Cronbach’s alpha = 0.903) and amotivation (McDonald’s omega = 0.891; Cronbach’s alpha = 0.888). The items of the MWMS start with a stem question (“Why do you or would you put effort into your current job?”) followed by response options. Intrinsic motivation and amotivation were each assessed with three items (e.g., “Because I have fun doing my job”; “I don’t, because I really feel that I’m wasting my time at work”). All items were rated from 1 (“strongly disagree”) to 5 (“strongly agree”). Work motivation was assessed in Sample 1.

#### Antidepressant intake

Antidepressant intake was assessed with the following item: “Are you currently taking medication for depression?” Response options were “yes,” “no,” and “I am currently taking medication, but I am not sure if it is medication for depression.” Antidepressant intake was assessed in Samples 1 and 3.

#### Attribution-free depressive symptoms

Attribution-free depressive symptoms were assessed with the depression subscale of the Hospital Anxiety and Depression Scale (HADS-D)^44^. A sample item is: “I still enjoy the things I used to enjoy.” All items were rated from 1 (“strongly disagree”) to 5 (“strongly agree”). In this study, McDonald’s omega was 0.851 and Cronbach’s alpha was 0.847. The HADS is a well-established instrument in depression research^45,46^. Attribution-free depressive symptoms were assessed in Sample 2.

#### Physical assault and verbal abuse at work

Participants indicated whether they had been (a) physically assaulted and (b) verbally abused in the context of their work over the past six months. Response options were “yes,” “no,” and “I’m not sure.” Physical assault and verbal abuse at work were assessed in Sample 2.

#### Workplace ostracism

Workplace ostracism was assessed with the Ostracism Short Scale (OSS)^47^. The OSS comprises four items (e.g., “Others treated me as if I wasn’t there”). Each item was rated from 1 for “never” to 5 for “always.” McDonald’s omega was 0.856. Cronbach’s alpha was 0.853. Workplace ostracism was assessed in Sample 2.

#### Socioeconomic optimism

We assessed socioeconomic optimism with the following item: “Are you optimistic about the socioeconomic future of Norway in the decades to come?” The item was rated from 1 (“not optimistic at all”) to 5 (“extremely optimistic”). Socioeconomic optimism was assessed in Sample 2.

#### Workplace bullying

Workplace bullying was assessed with the short version of the Negative Acts Questionnaire (S-NAQ)^48^. The measure is made up of nine items (e.g., participants were asked about whether they had to endure “practical jokes carried out by people you do not get al.ong with”). Each item was rated on a 5-point scale from “never” to “daily.” McDonald’s omega was 0.921. Cronbach’s alpha was 0.920. Workplace bullying was assessed in Sample 3.

### Data analysis

**Fig. 1:**
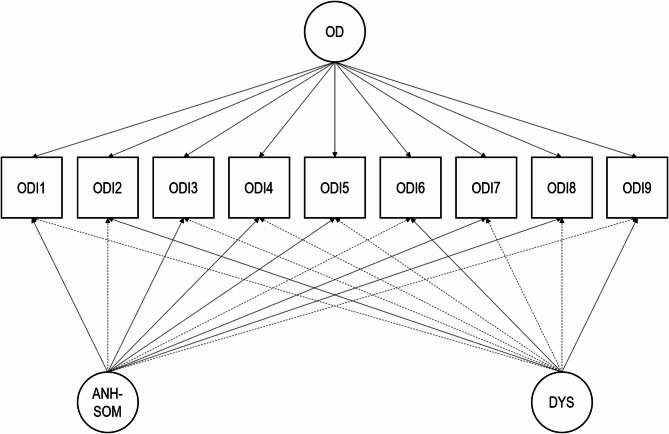
Exploratory structural equation modeling bifactor structure under examination. A target rotation is used. Solid lines are indicative of target loadings; dotted lines were used for the remaining loadings. Two specific factors are considered on account of the anhedonic-somatic and dysphoric symptom items populating the Occupational Depression Inventory. OD: general Occupational Depression factor; ANH-SOM: Anhedonic-Somatic specific factor; DYS: Dysphoric specific factor. ODI1: anhedonia; ODI2: depressed mood; ODI3: sleep alterations; ODI4: fatigue/loss of energy; ODI5: appetite alterations; ODI6: feelings of worthlessness; ODI7: cognitive impairment; ODI8: psychomotor alterations; ODI9: suicidal ideation.

Consistent with previous research^10,13,18–20,23–26^, we examined the dimensionality of the ODI based on ESEM bifactor analysis^27,49^. A bifactor model partitions the total covariance among a scale’s items into a general factor underlying all items and specific factors explaining additional covariance not captured by the general factor. We treated the ODI’s items as ordinal and used the weighted least squares–mean and variance adjusted–(WLSMV) estimator. We employed a target rotation, allowing for theoretically driven item-factor specifications. We considered two specific factors in addition to the general factor–Occupational Depression (Fig. 1). Two specific factors were extracted on the basis of the ODI’s “anhedonic-somatic” items (Items 1, 3, 4, 5, 7, and 8) and “dysphoric” items (Items 2, 6, and 9). The omega hierarchical and explained common variance (ECV) statistics were computed. Omega hierarchical is an index of the proportion of total score variance that can be attributed to the general factor, after accounting for the variance explained by the specific factors and error. The ECV index estimates the proportion of the common variance (i.e., variance shared among items) that is attributable to the general factor, with values ≥ 0.80 suggesting essential unidimensionality^13,27^. We relied on the following indices to estimate the fit of the model: the Root Mean Square Error of Approximation (RMSEA); the Comparative Fit Index (CFI); the Tucker-Lewis Index (TLI); and the Standardized Root Mean Square Residual (SRMR). We considered acceptable model fit to be indicated by an RMSEA value ≤ 0.08, a CFI value ≥ 0.95, a TLI value ≥ 0.95, and a SRMR value ≤ 0.08^10,13^. We conducted these analyses in Mplus 8.7^50^.

We gauged the ODI’s scalability using the Mokken package version 3.0.6^51^ in R version 4.2.0^52^. Scalability estimates the extent to which a scale’s items hierarchically align on a single dimension. Scalability is indexed by Loevinger’s *H* coefficients. The following benchmarks are generally utilized^51^: scalability is considered weak if 0.30 ≤ *H* < 0.40, moderate if 0.40 ≤ *H* < 0.50, and strong if *H* ≥ 0.50. A scale-level *H* coefficient < 0.30 suggests that the examined scale cannot be regarded as unidimensional. Pairwise *H* coefficients should be > 0; item-level *H* coefficients should be > 0.30. We complementarily relied on the automated item selection procedure (AISP), which identifies unidimensional scales within a set of items by grouping together the items that meet a specified minimum scalability threshold. As recommended, we tested thresholds in ascending increments of 0.05, starting at 0.30, and stopped when the scale fragmented or an item became unscalable^51,53^. Monotonicity violations were examined in terms of their presence and severity. We computed McDonald’s omega, Cronbach’s alpha, Guttman’s lambda-2, and the Molenaar-Sijtsma statistic to index the ODI’s total-score reliability.

The ODI’s criterion validity was tested with the Pearson correlation and, in the case of work addiction, with the Pearson correlation and Welch’s analysis of variance. To estimate convergent validity and discriminant validity, we examined the ODI in relation to the HADS-D using the Pearson correlation and ESEM bifactor analysis with target rotation. In the bifactor analysis, we extracted two specific factors–one linked to the ODI’s items and the other linked to the HADS-D’s items. We treated all items as ordinal and employed the WLSMV estimator. A similar analytical approach has been employed in several ODI studies^20,24,26^.

Finally, we examined the invariance of the ODI’s structure across samples (Sample 1 vs. Sample 2 vs. Sample 3), sexes (males vs. females), and age segments (18–34 [early career] vs. 35–49 [mid-career] vs. 50+ [late career]). We estimated the equivalence of (a) overall factorial structures–configural invariance, (b) factor loadings–metric invariance, and (c) item thresholds–scalar invariance^54^. Researchers have come up with various threshold values for flagging violations of measurement invariance. We considered a conservative threshold of 0.005 for identifying problematic changes in RMSEA, CFI, TLI, and SRMR^13,54^.

## Results

### Factorial validity and dimensionality

In Sample 1, the specified ESEM bifactor structure exhibited a satisfactory fit: RMSEA = 0.000; CFI = 1.000; TLI = 1.000; SRMR = 0.007; *χ²* (12) = 9.459. All ODI items loaded substantially and preponderantly on the general factor (*M* = 0.854, *SD* = 0.068). While the Dysphoric bifactor was well-delineated, the Anhedonic-Somatic bifactor retained only a limited amount of specificity beyond the variance explained by the general factor. The coefficient omega hierarchical had a value of 0.920, and the general factor accounted for 90.0% of the common variance extracted, suggesting that the ODI met the requirements for essential unidimensionality.

In Samples 2 and 3, the specified ESEM bifactor structure represented an over-factored solution. We thus switched to a fully unidimensional confirmatory factor analytic (CFA) model–all ODI items were allowed to load on a single factor with no secondary dimensions considered. The model showed a satisfactory fit in both samples. In Sample 2, we found the following fit indices: RMSEA = 0.080; CFI = 0.983; TLI = 0.977; SRMR = 0.063; *χ²* (27) = 110.164. All ODI items displayed substantial factor loadings (*M* = 0.782, *SD* = 0.062). In Sample 3, the fit indices were as follows: RMSEA = 0.052; CFI = 0.995; TLI = 0.993; SRMR = 0.036; *χ²* (27) = 52.436. Once more, all ODI items displayed substantial factor loadings (*M* = 0.841, *SD* = 0.037).

### Scalability

The results are summarized in Table 3. The ODI displayed strong scalability in each of the three samples: 0.577 ≤ scale-level *H*s ≤ 0.706. All item-level and pairwise *H*s were consistent with this observation. The AISP further supported the ODI’s scalability; a single scale comprising all nine items was still discernible at thresholds ranging from 0.500 to 0.600, depending on the sample. In all samples, the most widely endorsed item was fatigue/loss of energy and the least widely endorsed item was suicidal ideation. No monotonicity violations were detected.

**Table 3:** Scalability and reliability analyses of the Occupational Depression Inventory (ODI).

Items	Sample 1 (*N* = 382)			Sample 2 (*N* = 485)			Sample 3 (*N* = 353)		
	H	SE	95% CI	H	SE	95% CI	H	SE	95% CI
ODI1 (anhedonia)	0.718	0.031	[0.658, 0.778]	0.615	0.025	[0.565, 0.665]	0.705	0.032	[0.643, 0.767]
ODI2 (depressed mood)	0.712	0.029	[0.655, 0.770]	0.562	0.028	[0.507, 0.618]	0.672	0.034	[0.606, 0.739]
ODI3 (sleep alterations)	0.717	0.032	[0.654, 0.781]	0.582	0.030	[0.523, 0.641]	0.669	0.035	[0.601, 0.737]
ODI4 (fatigue/loss of energy)	0.750	0.029	[0.694, 0.806]	0.660	0.024	[0.613, 0.708]	0.667	0.035	[0.599, 0.735]
ODI5 (appetite alterations)	0.690	0.032	[0.627, 0.753]	0.530	0.036	[0.460, 0.600]	0.632	0.038	[0.558, 0.706]
ODI6 (feelings of worthlessness)	0.685	0.031	[0.625, 0.745]	0.513	0.033	[0.449, 0.577]	0.617	0.039	[0.541, 0.692]
ODI7 (cognitive impairment)	0.735	0.029	[0.677, 0.792]	0.610	0.028	[0.556, 0.665]	0.679	0.032	[0.616, 0.743]
ODI8 (psychomotor alterations)	0.724	0.031	[0.664, 0.785]	0.569	0.031	[0.510, 0.629]	0.638	0.037	[0.565, 0.711]
ODI9 (suicidal ideation)	0.565	0.056	[0.454, 0.675]	0.508	0.068	[0.374, 0.642]	0.648	0.053	[0.544, 0.752]
OD (occupational depression)	0.706	0.027	[0.654, 0.759]	0.577	0.025	[0.528, 0.626]	0.659	0.031	[0.598, 0.720]
Automated Item Selection Procedure*	0.550			0.500			0.600		
Monotonicity violations	0			0			0		
McDonald’s omega	0.938			0.900			0.922		
Cronbach’s alpha	0.933			0.893			0.920		
Guttman’s lambda-2	0.938			0.900			0.922		
Molenaar-Sijtsma statistic	0.939			0.903			0.930		

H Loevinger’s scalability coefficient; *SE*: standard error; 95% CI: 95% confidence interval. All pairwise *H* coefficients were (substantially) greater than 0. * We report the highest threshold at which a single scale comprising all items could be identified; thresholds were tested in ascending increments of 0.050, starting at 0.300.

### Total-score reliability

The ODI exhibited high total-score reliability in all samples (Table 3). McDonald’s omega ranged from 0.900 to 0.938; Cronbach’s alpha, from 0.893 to 0.933; Guttman’s lambda-2, from 0.900 to 0.938; and the Molenaar-Sijtsma statistic, from 0.903 to 0.939.

### Criterion validity

Zero-order correlations among the main variables of interest are presented in Tables 4, 5 and 6. In all samples, occupational depression correlated positively with turnover intention and sick leave and negatively with age. Correlations of occupational depression with job promotion, though in the expected direction (i.e., negative), were small and statistically nonsignificant.

**Table 4:** Zero-order correlations among the main variables of interest in Sample 1.

		Theoretical range	M	SD	*N*	2.	3.	4.	5.	6.	7.	8.	9.	10.
1.	Occupational depression	0–3	0.526	0.603	382	0.682	−0.389	0.465	0.289	−0.076	0.141	−0.080	−0.189	0.617
2.	Work addiction	1–5	2.134	0.770	382	–	**-0.159**	**0.242**	**0.207**	0.017	**0.159**	−0.042	**-0.190**	**0.359**
3.	Intrinsic motivation	1–5	3.654	0.951	382		–	**-0.726**	**-0.111**	**0.132**	−0.004	−0.032	**0.148**	**-0.445**
4.	Amotivation	1–5	1.889	0.943	382			–	**0.173**	**-0.125**	0.013	0.039	**-0.200**	**0.568**
5.	Sick leave	0/1	0.314	0.465	382				–	−0.030	**0.132**	−0.067	−0.091	**0.306**
6.	Job promotion	0/1	0.149	0.357	382					–	0.013	−0.052	**-0.122**	−0.108
7.	Antidepressant intake	0/1	0.064	0.244	377						–	−0.016	0.029	0.079
8.	Sex	0/1	0.491	0.501	381							–	**0.210**	−0.031
9.	Age	1–3*	–	–	382								–	**-0.209**
10.	Turnover intention	1–3**	–	–	244									–

Correlation coefficients that are statistically significant (*p* < .05) are shown in bold. Sex was coded 0 for women and 1 for men. For the other binary variables, 0 meant “absence” and 1 meant “presence.” With respect to variable 7, individuals who responded “I’m not sure” were excluded from the analyses. One individual did not respond to the sex item. * Age was categorized as follows: 18–34 (early career); 35–49 (mid-career); and 50+ (late career). ** 138 participants reported not having experienced work-related depressive symptoms and were excluded from the analyses that involved turnover intention. *M*: mean; *SD*: standard deviation.

**Table 5:** Zero-order correlations among the main variables of interest in Sample 2.

		Theoretical range	M	SD	*N*	2.	3.	4.	5.	6.	7.	8.	9.	10.	11.
1.	Occupational depression	0–3	0.631	0.581	485	0.661	0.091	0.224	0.264	−0.071	0.415	−0.307	−0.186	−0.128	0.485
2.	General depression	1–5	2.093	0.658	485	–	0.017	0.089	**0.250**	**−0.098**	**0.412**	**-0.317**	−0.078	**-0.107**	**0.430**
3.	Physical assault at work	0/1	0.071	0.257	481		–	**0.330**	0.024	−0.055	0.013	−0.025	−0.013	**-0.192**	−0.019
4.	Verbal abuse at work	0/1	0.288	0.453	469			–	0.063	−0.021	**0.185**	**-0.219**	−0.022	**-0.176**	**0.130**
5.	Sick leave	0/1	0.231	0.422	485				–	−0.036	**0.152**	**-0.149**	**-0.132**	0.053	**0.166**
6.	Job promotion	0/1	0.196	0.397	485					–	−0.048	0.053	0.039	**-0.124**	**-0.101**
7.	Workplace ostracism	1–5	1.593	0.689	485						–	**-0.244**	−0.020	0.025	**0.337**
8.	Socioeconomic optimism	1–5	2.456	0.858	485							–	0.070	0.000	**-0.260**
9.	Sex	0/1	0.315	0.465	480								–	0.054	**-0.128**
10.	Age	1–3*	–	–	485									–	−0.096
11.	Turnover intention	1–3**	–	–	387										–

Correlation coefficients that are statistically significant (*p* < .05) are shown in bold. Sex was coded 0 for women and 1 for men. For the other binary variables, 0 meant “absence” and 1 meant “presence.” Regarding variables 3 and 4, individuals who responded “I’m not sure” were excluded from the analyses. Five individuals did not respond to the sex item. * Age was categorized as follows: 18–34 (early career); 35–49 (mid-career); and 50+ (late career). ** 98 participants reported not having experienced work-related depressive symptoms and were excluded from the analyses that involved turnover intention. *M*: mean; *SD*: standard deviation.

**Table 6:** Zero-order correlations among the main variables of interest in Sample 3.

		Theoretical range	M	SD	*N*	2.	3.	4.	5.	6.	7.	8.
1.	Occupational depression	0–3	0.579	0.605	353	0.537	0.187	−0.076	0.226	−0.137	−0.182	0.564
2.	Workplace bullying	1–5	1.399	0.584	353	–	**0.230**	0.023	**0.198**	0.074	−0.064	**0.370**
3.	Sick leave	0/1	0.297	0.458	353		–	−0.053	0.099	−0.086	0.024	**0.220**
4.	Job promotion	0/1	0.153	0.360	353			–	**0.150**	0.080	**-0.166**	**-0.116**
5.	Antidepressant intake	0/1	0.077	0.268	349				–	−0.031	−0.073	0.066
6.	Sex	0/1	0.480	0.500	352					–	0.098	**-0.140**
7.	Age	1–3*	–	–	353						–	**-0.116**
8.	Turnover intention	1–3**	–	–	294							–

Correlation coefficients that are statistically significant (*p* < .05) are shown in bold. Sex was coded 0 for women and 1 for men. For the other binary variables, 0 meant “absence” and 1 meant “presence.” Regarding variable 5, individuals who responded “I’m not sure” were excluded from the analyses. One individual did not respond to the sex item. * Age was categorized as follows: 18–34 (early career); 35–49 (mid-career); and 50+ (late career). ** 59 participants reported not having experienced work-related depressive symptoms and were excluded from the analyses that involved turnover intention. *M*: mean; *SD*: standard deviation.

Analyses of Sample 1 indicated that occupational depression correlated negatively with intrinsic motivation and positively with amotivation, antidepressant intake, and work addiction. ODI scores were more than two standard deviations higher among workaholics (*M* = 1.514, *SD* = 0.825) than among non-workaholics (*M* = 0.436, *SD* = 0.489), Welch’s *F*(1, 33.022) = 52.953, *p* < .001, Cohen’s *d* = 2.055 (95% CI = 1.665, 2.445). Occupational depression showed a small, statistically nonsignificant association with sex, with the direction of the association indicating higher scores among women. Analyses of Sample 2 indicated that occupational depression correlated negatively with socioeconomic optimism and positively with physical assault at work, verbal abuse at work, and workplace ostracism. Occupational depression showed a modest but statistically significant association with sex, with the direction of the association indicating higher scores among women. Analyses of Sample 3 indicated that occupational depression correlated positively with workplace bullying and antidepressant intake. Once more, the association with sex was modest but statistically significant and indicated higher scores among women.

### Convergent and discriminant validity

The specified ESEM bifactor structure showed a satisfactory fit: RMSEA = 0.059; CFI = 0.985; TLI = 0.977; SRMR = 0.031; *χ*^*2*^ (75) = 203.122. On the one hand, all ODI and HADS-D items loaded substantially on the general factor (*M* = 0.659, *SD* = 0.094), a finding suggestive of a degree of convergent validity. The mean factor loading on the general factor was 0.729 for HADS-D items (*SD* = 0.099) and 0.604 for ODI items (*SD* = 0.041). On the other hand, the ODI bifactor was well-delineated, and the explained common variance index linked to ODI items had a value of only 0.576, suggesting a degree of discriminant validity. The HADS-D bifactor was weaker, though not insubstantial. Consistent with our factor-analytic findings (further detailed in Supplemental Material 2), scores on the ODI correlated 0.661 (*p* < .001) with scores on the HADS-D (Table 5).

### Measurement invariance

We found evidence for complete measurement invariance across samples, sexes, and age segments. Across all models, as constraints were added, RMSEA never increased, CFI never decreased by more than 0.003, TLI never decreased, and SRMR never increased by more than 0.003. The results are summarized in Table 7.

**Table 7:** Measurement invariance analysis of the Occupational Depression Inventory.

	χ^2^	df	RMSEA	ΔRMSEA	CFI	ΔCFI	TLI	ΔTLI	SRMR	ΔSRMR
Across samples										
Configural model	290.062	81	0.080	–	0.989	–	0.985	–	0.036	–
Metric model	351.512	97	0.080	0.000	0.986	−0.003	0.985	0.000	0.039	0.003
Scalar model	351.585	131	0.064	−0.016	0.988	0.002	0.990	0.005	0.041	0.002
Across sexes										
Configural model	257.677	54	0.079	–	0.987	–	0.983	–	0.033	–
Metric model	292.595	62	0.078	−0.001	0.986	−0.001	0.984	0.001	0.034	0.001
Scalar model	292.673	79	0.067	−0.011	0.987	0.001	0.988	0.004	0.035	0.001
Across age segments										
Configural model	303.669	81	0.082	–	0.987	–	0.983	–	0.036	–
Metric model	335.565	97	0.078	−0.004	0.986	−0.001	0.984	0.001	0.038	0.002
Scalar model	325.992	131	0.060	−0.018	0.989	0.003	0.991	0.007	0.039	0.001

χ^2^: chi-square; df: degrees of freedom; RMSEA: Root Mean Square Error of Approximation; CFI: Comparative Fit Index; TLI: Tucker-Lewis Index; SRMR: Standardized Root Mean Squared Residual; Δ: delta (change in).

## Discussion

This study examined the psychometric and structural properties of the Norwegian version of the ODI. We capitalized on three samples of employed individuals and relied on advanced statistical techniques. We investigated the dimensionality, scalability, total-score reliability, criterion validity, convergent validity, discriminant validity, and measurement invariance of the measure. Our findings supported our hypotheses.

### Main findings

Our results bearing on the dimensionality of the ODI comport with those obtained in other countries and languages^10,13,18–20,23–26^. The measure exhibited essential, and even strict, unidimensionality, supporting the use of the instrument’s total score. Consistent with these findings, the ODI displayed strong scalability and a clear gradient of symptom endorsement, from more common symptoms such as fatigue/loss of energy to rarer and more severe symptoms such as suicidal ideation. While suicidal ideation was the least endorsed item, it demonstrated strong scalability–*H*s > 0.50–in all samples. This suggests that, when individuals do endorse the item, their responses are consistent with a broader pattern of high occupational depression severity, supporting the item’s coherence within the overall scale. Items with such characteristics can serve as markers for identifying individuals at clinical risk. On this basis, the ODI’s suicidal ideation item has been regarded as a *sentinel item*^13^.

The total-score reliability of the instrument was high irrespective of the index employed. It is of note that total-score reliability can be inflated by the repetition of synonymous items in measures of interest^38,55^. It is no surprise that a measure may show internal consistency if the questions it includes are redundant and interchangeable. Such an inflation phenomenon is unlikely with the ODI because each of the nine items of the scale refers to a specific symptom, with virtually no synonymy^10,13^. The associations across the items more probably reflect clinically meaningful bonds among the symptoms assessed.

Measurement invariance held across samples, sexes, and age segments, suggesting that the ODI was understood and employed similarly by people from these different groups. Measurement invariance is a necessary condition for conducting valid comparisons across groups of interest. Without measurement invariance, one cannot ascertain whether observed differences between groups are due to actual differences in the construct being measured or to the measure working differently across the groups examined^56^. Our findings are consistent with those from other countries in which the ODI has been tested^13,18–20,24,57^, including neighboring Sweden^25^.

As anticipated, the ODI showed a balance of convergent and discriminant validity vis-à-vis the HADS-D, a measure of depressive symptoms that does *not* reference work. Similar findings have been obtained in the past, using the HADS-D but also different depression scales, including the Center for Epidemiologic Studies Depression scale, the depression subscale of the Depression Anxiety Stress Scales-21, and the PHQ-9^10,20,24,25^. These findings align with the fact that the ODI focuses on *work-attributed* symptoms rather than *attribution-free* symptoms^10^.

In line with the research conducted on general depression^29,37^, we found that occupational depression correlated (a) positively with work addiction, amotivation toward work, sick leave, antidepressant intake, physical assault at work, verbal abuse at work, workplace ostracism, workplace bullying, and turnover intention and (b) negatively with intrinsic motivation toward work and socioeconomic optimism. Contrary to expectations, no association was found with job promotion. This result is unlikely to be linked to a low prevalence of job promotion in our samples. Indeed, the prevalence of job promotion ranged from 14.9% to 19.6%, representing a total of 206 participants. Interestingly, a similar result was obtained in a study that validated the ODI in Italy^19^. Overall, the present study supports the ODI’s criterion validity based on a broad array of variables, including important job stressors such as workplace ostracism and workplace bullying. We encourage future studies to adopt longitudinal designs to examine the predictive power of the ODI in relation to key outcomes such as actual turnover, long-term absenteeism, and clinical diagnoses of depressive disorders.

### Strengths and limitations

The use of three different samples enabled us to replicate core findings within a single study. Although replication is a cornerstone of scientific research, it has faced challenges in psychological research. Within-study replication can help address these challenges. Another strength of our study is the reliance on advanced analytical techniques such as ESEM and Mokken scaling. Our analytical strategy permitted us to examine the ODI at several levels of resolution, including granular ones. Triangulating evidence from different types of analyses strengthens confidence in the study’s findings.

As for its limitations, our study had a cross-sectional design, which prevented us from examining properties such as temporal measurement invariance. The use of a cross-sectional design additionally bore on our investigation of criterion validity. We were able to examine concurrent validity rather than predictive validity. In addition, more measures of attribution-free depressive symptoms could have been included for comparisons with the ODI. Furthermore, although person-reported information is highly valuable for clinical research^58^, it would have been an added advantage to consider objective indicators of health and performance^20–22^. With regard to health, allostatic load indices, hospital or physician visits, and absenteeism records could serve as informative indicators; regarding performance, metrics of work output and neuropsychological testing would be useful. Finally, we relied on several single-item measures, a practice traditionally regarded as suboptimal. There is evidence, however, that such measures can perform as well as their multi-item counterparts^59^.

### Conclusions

We found the ODI to exhibit excellent psychometric and structural properties in Norway. Our results showed high consistency across samples and analyses. All in all, our findings support the use of the ODI in the Norwegian occupational health context. From a broader perspective, the present study adds to an extended corpus of research indicating that the ODI constitutes a robust instrument. The ODI may help occupational health specialists assess and address job-related distress effectively.

## Supplementary Information

Below is the link to the electronic supplementary material.

Supplementary Material 1

Supplementary Material 2

## Data Availability

The data that were used in this study are available upon reasonable request from the corresponding author (RB).
