# Supplementary material for: The Occupational Depression Inventory performs well in Norway
Source: Sci Rep. 2026 May 8;16:21175. doi: 10.1038/s41598-026-52564-x (PMC13342518; doi:10.1038/s41598-026-52564-x)
Supplement: Supplementary file 1 — Supplementary Material 1 [file 41598_2026_52564_MOESM1_ESM.pdf]

Supplementary Material 2. Exploratory structural equation modeling bifactor analysis of the Occupational Depression Inventory (ODI) and the depression subscale of the Hospital Anxiety and Depression Scale (HADS-D).

Item	GF	SF1	SF2	I-ECV	S-ECV	ECV
ODI1	**0.613**	**0.576**	-0.136	0.518	0.576	0.701
ODI2	**0.670**	**0.433**	-0.164	0.677		
ODI3	**0.530**	**0.579**	0.003	0.456		
ODI4	**0.614**	**0.587**	-0.004	0.522		
ODI5	**0.605**	**0.436**	0.164	0.628		
ODI6	**0.618**	**0.382**	-0.037	0.722		
ODI7	**0.559**	**0.649**	0.007	0.426		
ODI8	**0.593**	**0.544**	-0.021	0.543		
ODI9	**0.633**	0.293	0.135	0.795		
HADS-D1	**-0.800**	-0.003	0.274	0.894	0.866	
HADS-D2	**-0.747**	0.096	**0.358**	0.803		
HADS-D3	**-0.807**	-0.012	**0.359**	0.834		
HADS-D4	**0.580**	0.206	-0.047	0.883		
HADS-D5	**0.630**	-0.103	**0.427**	0.673		
HADS-D6	**-0.846**	0.056	0.022	0.994		
HADS-D7	**-0.696**	0.035	0.019	0.997		

Notes. N = 485 (Sample 2). Loadings ≥ 0.30 are bolded. GF: general Depression factor; SF1: specific “attribution-free depressive symptoms” factor; SF2: specific “work-attributed depressive symptoms” factor; ECV: Explained Common Variance; S-ECV: scale-level ECV; I-ECV: item-level ECV.
