# Supplementary material for: The Occupational Depression Inventory performs well in Norway
Source: Sci Rep. 2026 May 8;16:21175. doi: 10.1038/s41598-026-52564-x (PMC13342518; doi:10.1038/s41598-026-52564-x)
Supplement: Supplementary file 2 — Supplementary Material 2 [file 41598_2026_52564_MOESM2_ESM.pdf]

KARTLEGGINGSVERKTØY FOR YRKESRELATERT DEPRESJON (KYD)

INNLEDENDE INSTRUKSJONER TIL RESPONDENTER

Følgende utsagn omhandler innvirkningen ditt arbeid kan ha hatt på deg.

Vennligst les hvert utsagn og indiker hvor ofte du opplevde de nevnte problemene i løpet av de TO SISTE UKENE. Bruk den oppgitte skalaen for å svare:

0 = aldri eller nesten aldri

1 = kun noen få dager

2 = mer enn halvparten av dagene

3 = nesten hver dag

Her er et eksempel:

«Jeg følte meg engstelig på grunn av jobben min.»

• Hvis du IKKE følte deg engstelig på grunn av jobben din, velg **0**.

• Hvis du følte deg engstelig av grunner som du IKKE mener er relatert til jobben din (personlige problemer, ekteskapelige problemer, familieproblemer, helseproblemer osv.) velger du også **0**.

• Hvis du følte deg engstelig, men du vet ikke hvorfor, velg **0** igjen.

• Hvis det er åpenbart for deg at JOBBEN DIN fikk deg til å føle deg engstelig, velg **1**, **2** eller **3** for å indikere hvor ofte det hendte.

Du kan nå fullføre spørreskjemaet.

KARTLEGGINGSVERKTØY FOR YRKESRELATERT DEPRESJON (KYD)

Pasientens navn: .... Dato: ....

**Indiker hvor ofte du opplevde de nevnte problemene nedenfor i løpet av de to siste ukene.**	*Aldri eller nesten aldri*	*Kun noen få dager*	*Mer enn halvparten av dagene*	*Nesten hver dag*
1. Mitt arbeid var så stressende at jeg ikke kunne glede meg over ting jeg vanligvis liker å gjøre.	**0**	**1**	**2**	**3**
2. Jeg følte meg deprimert på grunn av jobben min.	**0**	**1**	**2**	**3**
3. Stress relatert til jobben førte til søvnproblemer (jeg hadde vanskelig for å sovne eller sove uforstyrret, eller jeg sov mye mer enn vanlig).	**0**	**1**	**2**	**3**
4. Jeg følte meg utmattet på grunn av arbeidet mitt.	**0**	**1**	**2**	**3**
5. Jeg følte at appetitten min ble forstyrret på grunn av jobbstress (jeg mistet appetitten min, eller det motsatte, jeg spiste for mye).	**0**	**1**	**2**	**3**
6. Min opplevelse på jobb fikk meg til å føle meg mislykket.	**0**	**1**	**2**	**3**
7. Jobben min stresset meg så mye at jeg hadde problemer med å fokusere på det jeg gjorde (f.eks. å lese en avisartikkel) eller å tenke klart (f.eks. å ta beslutninger).	**0**	**1**	**2**	**3**
8. Som et resultat av jobbstress følte jeg meg rastløs, eller det motsatte, alt gikk saktere–for eksempel i måten jeg beveget meg eller snakket på.	**0**	**1**	**2**	**3**
9. Jeg tenkte at jeg ville heller være død enn å fortsette i denne jobben.	**0**	**1**	**2**	**3**

TOTALSKÅR: ....

Dersom du har støtt på minst noen av problemene nevnt ovenfor, fører disse problemene til at du vurderer å slutte i din nåværende jobb eller stilling?

☐ Ja ☐ Nei ☐ Jeg vet ikke

Occupational Depression Inventory: SPSS syntax for a provisional diagnosis of occupational depression

```
compute DEP = 0.
do if ODI1 ge 3 or ODI2 ge 3.
count DEP = ODI3 (3)
ODI4 (3)
ODI5 (3)
ODI6 (3)
ODI7 (3)
ODI8 (3)
ODI9 (1,2,3).
end if.

if ODI1 ge 3 DEP = DEP + 1.
if ODI2 ge 3 DEP = DEP + 1.

compute DIAG = 0.
if DEP ge 5 DIAG = 1.
```

Note. The nine items of the Occupational Depression Inventory (ODI) are coded ODI1 to ODI9. Make sure ODI scores are coded from 0 to 3.

Items

ODI1: anhedonia

ODI2: depressed mood

ODI3: sleep alterations

ODI4: fatigue/loss of energy

ODI5: appetite alterations

ODI6: feelings of worthlessness

ODI7: cognitive impairment

ODI8: psychomotor alterations

ODI9: suicidal ideation
